# Telemedicine in non-communicable chronic diseases care during the COVID-19 pandemic: exploring patients' perspectives

**DOI:** 10.3389/fpubh.2023.1270069

**Published:** 2023-09-25

**Authors:** Fatema Habbash, Aamal Rabeeah, Zainab Huwaidi, Hiba Abuobaidah, Jumana Alqabbat, Fatema Hayyan, Amer Almarabheh, Hind Al Sindi, Afif Ben Salah

**Affiliations:** ^1^Department of Family and Community Medicine, Arabian Gulf University, Manama, Bahrain; ^2^Department of Family Medicine, University Medical Center King Abdullah Medical City Bahrain, Manama, Bahrain; ^3^Primary Health Care, Manama, Bahrain; ^4^Institute Pasteur de Tunis, Department of Medical Epidemiology, Tunis, Tunisia

**Keywords:** telemedicine, non-communicable chronic diseases, COVID-19, patients' perspectives, satisfaction, willingness

## Abstract

**Purpose:**

This study aimed to explore challenges facing patients using Telemedicine consultations in non-communicable chronic disease clinics in primary care settings and to evaluate their satisfaction and willingness to use this service in the future.

**Methods:**

This is an analytical cross-sectional study enrolling participants who were randomly selected from representative primary care centers in Bahrain and providing Telemedicine consultations. A semi-structured questionnaire permitted data collection using telephone interviews.

**Results:**

A total of 251 individuals participated in the study of whom the majority were Bahraini (90.04%), and the mean age was 54.48 ± 10.78 years. Most of the participants 231 (92.03%) were satisfied with the Telemedicine consultation while only 142 (56.80%) were willing to use this service in the future. The main perceived challenges related to Teleconsultations were the lack of physical examination, inadequate time of TM consultation, fear of medical errors, and lack of privacy. The willingness to use TM consultation in the future was mainly determined by the degree of comfort to tell private information (*p* < 0.01) and to less extent the ease of the communication tool (*p* = 0.005) on multivariate analysis.

**Conclusion:**

TM consultations could be a good complement to conventional consultation formats in the future. The sustainability of this innovative healthcare delivery tool requires addressing acceptability by users, ease of use, patient-centeredness, and technological advances to ensure privacy.

## Introduction

Telemedicine (TM) is defined as “the use of electronic information and telecommunications technologies to support and promote long-distance clinical health care, patient and professional health-related education, public health, and health administration” (1). TM involves remote patient monitoring, mobile health applications, and more traditional modes of communication such as text, email, voice, and video calls (2).TM services benefit patients and medical providers by reducing disease exposure, increasing healthcare accessibility, and allowing for more efficient use of hospital resources (3).

The burden of non-communicable chronic diseases (NCDs) remains a global public health challenge, resulting in high morbidity, mortality, and cost (4, 5). In 2019, NCDs were responsible for 74% of global deaths, with most Gulf Cooperation Council countries exceeding this global average (6). Over the past several years, Bahrain has made significant strides in preventing and controlling NCDs (6, 7). Nonetheless, their prevalence is increasing and NCDs such as cardiovascular disease, diabetes, cancer, and chronic lung disease currently account for nearly 75% of all deaths in Bahrain and ~one in five adults dies from NCDs-related problems before the age of 70 (6). Furthermore, poor patient adherence increases NCD-related mortality and morbidity, which creates a burden on healthcare utilization and costs (7). Poor adherence to drugs and follow-up visits was exacerbated during the COVID-19 pandemic due to restrictions in accessibility to health settings for patients with NCDs at the global level (8). Indeed, NCDs-related mortality and morbidity could be reduced provided an optimal follow-up and implementation of preventive measures as well as limiting exposure to SARS-COV2 in the hospital settings (9). Integrating NCDs care into primary care is a cost-effective, affordable, and equitable paradigm of care that has the potential to reduce morbidity and mortality from NCDs (10).

The COVID-19 pandemic prompted the medical community to integrate TM to avoid disruption of patient care throughout the pandemic including NCDS preventive and curative services (11).

While there are many barriers to using TM, evaluating patients' satisfaction and their experience is clearly important to ensure compliance and sustainable implementation (12). Studies conducted in various contexts worldwide revealed an overall high level of satisfaction with TM services among patients and healthcare providers during the COVID-19 pandemic and reported willingness to continue its use after the pandemic (12–21).

Overall satisfaction with TM as a tool of health care delivery is likely to be influenced by the technology's perceived usefulness and the technical competence of the provider and patient (1), as well as patient's related factors such as age, gender, and level of education (12, 22). Measures of success include the provider's communication skills during the patient-doctor interaction as well as the degree of success in addressing the patient's concerns, and emotional needs during the TM consultation (23). Common reported benefits of TM were time savings from less traveling and waiting time, and improved accessibility, convenience, and cost efficiency (13). However, TM has been reported as being less suitable when a physical examination is needed, and the diagnosis was unknown (20).

A TM consultation was introduced as a pilot program in few primary care general clinics in the Kingdom of Bahrain in 2018. TM consultations for NCD care through phone format were launched in primary health care centers in the Kingdom of Bahrain during the COVID-19 period in March 2020, to ensure continuity of care and minimize the risk related to high mixing during face to face consultations. The NCD TM consultations were solely conducted by trained family physicians and NCD nurses were involved in calling patients the day before their appointments to remind them and to be prepared for the TM consultation the following day. Only follow-up patients in the NCD clinics received TM consultations, whereas new NCD patients had face-to-face consultations for their first appointment. Each NCD TM consultation was assigned 15 min, but the time can be extended according to the patient's condition and level of understanding. All physicians were providing a standardized content of TM consultations like the face-to-face following the NCD electronic medical record format, except the physical exam. Physicians were expected to document the consultation findings in NCD electronic medical record.

Despite its early implementation in the health care system, the long-term sustainability of TM needs to consider the patients' experience, expectations, and perspectives. To the best of our knowledge, this is the first study in the Kingdom of Bahrain to assess patients' satisfaction and challenges with TM consultations for NCD patients in primary care settings. It was conducted in the context of a quality improvement project to support the sustainable integration of TM as an additional mode of health care delivery to improve outreach, infection control, and reduce cost, particularly for older adults patients with NCDs in primary health care. The findings will pave the way to implement corrective action plans based on scientific evidence and the perspectives of end-users.

## Materials and methods

### Study design and variables

This is an analytical cross-sectional study. Outcome variables include overall satisfaction with TM consultation services for NCDs and the willingness to use it in the future. Independent variables comprise socio-demographic factors, co-morbidities, and patients' reported challenges and experiences with the service.

### Target population and sample size

The target population was patients with NCDs who had TM consultations during the period of June 2020 to December 2020 in the primary healthcare centers in the Kingdom of Bahrain. The study sample is calculated using the formula for the simple random sampling approach, where Z = 1.96, *P* = 0.5, E = margin of error = 0.05. The total estimated sample size was 285. Any patient who received the service during the allocated period, Bahraini and Non-Bahraini, 18 years or older were eligible to participate in the study. Patients who cannot speak Arabic or English or suffer from mental health problems or did not provide informed consent were excluded from the study. Participants were referred as having mental health problem if recorded in their electronic medical records having diagnosis of any mental health problem according to ICD-11.

### Sampling method and study tool

Primary care centers in Bahrain are grouped into five health regions, and one health center was selected randomly from each health region. A computer-generated random numbers list permitted the identification of potential volunteers from the database of NCD patients registered in the centers who were served by TM during the study period.

Potential volunteers were approached by telephone interviews conducted by five trained interviewers (research members) using a semi-structured questionnaire to seek their informed consent which was documented in the consent form and collect the required information. The questionnaire and related code book were tested, piloted, and validated by senior investigators before launching the phone survey. The interviews were not recorded for ethical reasons.

### Study variables

We defined two outcome variables, overall satisfaction with the TM consultation services for NCDs as well as the willingness to use it in the future. These two variables are expected to provide more valid patients' perspectives regarding this new healthcare delivery approach.

Explanatory factors included information collected in five sections regarding sociodemographic, comorbidities, participants' experience during the TM consultation, satisfaction regarding its different components, challenges faced while using the service, and recommendations for further improvement. The perceived challenges and recommendations were asked as an open question and provided answers are documented in a preconceived list from the literature (yes/no) if perceived as such. Non listed choices are documented under (other) option. Overall satisfaction with the TM consultation was evaluated from the responses to the question “How would you classify your satisfaction through your experience while using the service?” as “bad, neutral, good, very good, excellent” and from that we generated 3 categories “Not-satisfied, Neutral and Satisfied.”

### Statistical analysis

Categorical variables were presented as frequencies and percentages, and continuous variables were presented as means and standard deviations. From the responses frequency and percentages were calculated. Chi-square tests permitted to test the association between categorical variables. Crude Odds Ratios (COR) and the 95% confidence intervals (95% CI) allowed us to test the strength of pairwise association between the two outcome variables i.e., satisfaction and willingness to use Teleconsultations with other independent categorical variables. The logistic regression model was used to estimate the Adjusted odds ratios and their 95% CI to account for potential confounders. A *p*-value <0.05 was considered statistically significant. The data was entered into Excel and then exported to Statistical Package for the Social Sciences (SPSS) software version 28 for analysis.

### Ethical considerations

The study was conducted following the Declaration of Helsinki and the protocol was approved by the Research and Ethics Committee of the College of Medicine and Medical Sciences at Arabian Gulf University (approval number: E17-PI-11–21) and the primary healthcare research and ethics committee. Participants were provided information about the study and informed that participation was entirely voluntary and that refusing to participate would not affect future services. Prior to enrolling, all respondents provided informed consent by phone, which was documented by the interviewer in the consent form. Furthermore, all data collected was kept confidential and anonymous and was not used for any other purpose. Reports summarizing the findings are shared with the primary health care director to improve the service and patients received the contact number of the investigators in case they need feedback or to address any query.

## Results

###  Sample characteristics

A total of 251 individuals participated in the study. Slightly more than half of the study participants were female (52.40%). The mean age was 54.48 ± 10.78 years, and the majority of the participants were 41 years or older (89.11%). Most participants were Bahraini (90.04%), and 56.58% reported having a secondary school degree or more. The majority of the participants suffered from more than one NCD (76.49%) and the most reported comorbidities were diabetes (74.90%), hyperlipidemia (66.93%), and hypertension (63.75%). The sociodemographic and medical characteristics of the study participants are presented in Table 1.

**Table 1:** Sociodemographic characteristics and morbidity profile of the study sample (Total number of participants = 251).

**Characteristic**	***n* (%)**
**Age group**	Mean ± SD = 54.48 ± 10.78
20–40 Years	27 (10.89)
41–60 Years	149 (60.08)
> 60 Years Missing	72 (29.03) 3 (1.20)
**Gender**	
Male	119 (47.40)
Female	131(52.20)
Missing	1 (0.40)
**Nationality**	
Bahraini	226 (90.04)
Non-Bahraini	25 (9.96)
**Educational level**	
Non-educated	35 (13.94)
Elementary school education	29 (11.55)
Intermediate education	45 (17.93)
Secondary school education	83 (33.07)
University/Higher education	59 (23.51)
**Type of comorbidities**	
Hypertension	160 (63.75)
Diabetes	188 (74.90)
Dyslipidaemia	168 (66.93)
Thyroid Disease	37 (14.92)
Asthma	15 (6.10)
**Number of comorbidities**	
Patients with One disease	54 (21.51)
Patients with more than one disease	192 (76.49)
Missing	5 (1.99)

### Satisfaction with telemedicine consultation

Regarding the overall satisfaction with the TM consultations for NCD care, 231(92.03%) of the participants reported being satisfied, 13(5.18%) provided a neutral response, and only 7 (2.79%) reported being dissatisfied. More than a three-quarter of the participants 197 (78.49%) reported that the doctor introduced him/herself adequately and the history taking covered all the information related to their medical problem for 194 (77.29%) patients. Most of the participants agreed that the doctors gave a comprehensive explanation of their health condition (*n* = 227, 90.43%) and all their questions were properly addressed for 224 (89.24%) participants. Most of the study sample reported a good understanding of their problem after the TM consultation (*n* = 207, 82.50%), the treatment plan was shared and explained fully to them (*n* = 224, 89.24%) and the physician was able to answer all questions related to their medical condition (*n* = 224, 89.24%). Most of the participants reported that the consultation time was adequate (*n* = 201, 80.48%) and the communication tools were user-friendly (*n* = 200, 79.68%). In contrast, only 154 (61.35%) participants reported that they were comfortable sharing private information with the doctor through TM consultation.

### Willingness to use TM consultations in the future

In contrast with the overall satisfaction that was reported by most of the interviewed patients, only 142 (56.80%) participants stated that they are willing to use TM consultation services in the future. On the contrary, 74 participants (29.60%) reported that they are not willing to use the service in the future while 34 (13.60%) of the participants had a neutral opinion regarding its future use.

### Participants reported challenges and recommendations for the future use of telemedicine consultations

The most reported challenges by the participants were the lack of physical examination (41.20%), inadequate time for the TM consultation (14.50%), fear of medical errors (11.55%), and lack of privacy during the TM consultation (9.16%).

The main recommendations to improve the TM consultations were, to improve the physician-patient communication (28.40%), to use video calls (30.30%), and to increase the TM consultation time (21.10%).

### Determinants of willingness to use TM consultations in the future

Willingness to use TM consultations in the future provides the assessment of satisfaction from a different angle. It was significantly associated with, the level of comfort to tell private information A.O.R. = 7.608, 95% CI = [2.814–20.573] and to less extent the perceived ease of the communication tool A.O.R. = 9.336, 95% CI = [1.961–44.442] (Table 2).

**Table 2:** Determinants of the willingness to use telemedicine services.

**Factor**	C.OR^*^(95% CI)	***P*-value**	A.OR^**^(95% CI)	**P-value**
**Comfortable sharing private information**				
Do not agree	Ref.		Ref.	
Neutral	5.696 (2.068–15.688)	<0.001	3.740 (1.298–10.781)	0.015
Agree	12.521 (4.916–31.895)	<0.001	7.608 (2.814–20.573)	<0.001
**Communication tool was easy to use**				
Do not agree	Ref.		Ref.	
Neutral	5.000 (0.964–25.930)	0.055	4.869 (0.854–27.770)	0.075
Agree	17.643 (3.993–77.948)	<0.001	9.336 (1.961–44.442)	0.005

^*^C.OR, Crude odds ratio (Unadjusted odds ratio). ^**^A.OR, Adjusted odds ratio

## Discussion

During the COVID-19 pandemic, the front lines healthcare services including primary care clinics, were severely interrupted at the global level. Despite the initial unexpectedness many health systems responded timely using digital technologies during this crisis to ensure reasonable continuity of health care services. TM services have proven to be an essential component of the worldwide public health response, with the potential to serve as a “safety net” for patients when appropriately integrated (24–26).

In this study, we have evaluated patients' experience regarding TM consultations for NCD care which is a new technology in our context introduced during the pandemic. Data collected through phone interviews included patients' level of satisfaction with the service, as well as their willingness to use the service in the future. We realized that while 92.03% of interviewed volunteers reported their overall satisfaction, where only 56.80% were positive about its future use. We have also explored challenges faced by the patients as service receivers and factors that predict the willingness of its future use. This discrepancy could reflect some biases that led to an overestimation of satisfaction and justifies triangulating this measurement through the willingness to use this service in the future. Unsurprisingly, factors associated with the willingness to use the service were the extent to which the patient is comfortable disclosing private information, and the ease of telecommunication tools. The degree of comfort in sharing private information appeared to be the most important factor associated with the future use of TM consultations. These factors are in agreement with some reported challenges of TM consultations (19, 20). Indeed, the most reported challenges perceived by the study participants related to TM consultations were the lack of physical examination, inadequate time of TM consultation, fear of medical errors, and lack of privacy during TM consultations. In this study, there was no significant association between participants' sociodemographic data and comorbidities with the future willingness of using TM consultations. Although some studies revealed similar findings (13, 27), others have reported that younger age and higher educational level were associated with more willingness to use the service in the future, this finding could be explained by higher technology literacy among a relatively younger and better-educated study sample (19, 22, 28, 29).

The level of satisfaction and willingness to use TM consultations in the future reported by patients in this study is consistent with the findings in studies conducted in different contexts globally (2, 12, 13, 21, 22, 26, 28–32). Recent studies revealed high satisfaction with virtual consultations across a range of diseases and expressed a strong preference to continue to use virtual consultations as a complement to regular healthcare services even in the post-pandemic period (13, 20). Studies in Gulf Cooperation Council countries revealed similar positive attitudes and a general acceptance of TM consultations including those conducted for NCD patients (22, 26, 30–33). The high level of satisfaction and utilization of TM consultations during the COVID-19 period could be due to their effectiveness in maintaining the continuity of health care and overcoming the risks and challenges of in-person consultations (34, 35). TM is a new modality of healthcare delivery in our context and could be a good complement to the conventional consultation formats in the future if safety, effectiveness, patient-centeredness, efficiency, equity, and acceptability to users are warranted (12, 36). The long-term sustainability of TM should be considered and scaled up even beyond the COVID-19 period, particularly when we consider the dynamics of health applications in the digital vortex (13).

In addition to the reported challenges, participants recommended adding the video format to the telephone consultation for improving patient-doctor communication and connection, as well as increasing TM consultation time. Patients in various contexts worldwide have agreed that video consultations provided the same satisfaction as in-person visits and allowed them to explain properly their health problems (37–39). New advances in “augmented and virtual reality” are nowadays focused on research, development, and health care. They will be promising in creating a breakthrough in the acceptance of TM consultations by patients with the development of cheaper online communication devices (40). However, developers are still facing challenges meeting the needs of end users (41), which raises the importance of encouraging them as developers as well as promoting implementation research in the real context. Integrating novel Telecommunication tools and formats considering adequate time and better patient-doctor communication could ensure patient-centered care and improve the acceptability and future utilization of Teleconsultations.

As part of a quality improvement project in collaboration with the primary healthcare in the Kingdom of Bahrain, the present study will provide part of the scientific evidence needed for the new improvement plan of this consultation approach in the future.

Despite the originality of this study and the importance of its findings, it suffers from some limitations. First, the results might be influenced by the sociodemographic characteristics of the included participants. Second, the reported level of satisfaction and willingness to use TM consultations might be over-reported due to the lockdown imposed by the COVID-19 pandemic during the study. Despite enrolling participants from different regions in the Kingdom of Bahrain, a selection bias could exist as certain subgroups of participants were not able to use TM consultations due to different reasons that could be related to age, disability, economic status, technology literacy, and other factors were not represented in our study sample. A recall bias could exist since phone interviews were not conducted immediately after TM consultations. In addition, a social desirability bias could have increased the level of satisfaction given that interviewers are medical doctors, though not the treating ones. Previous exposure of participants to TM consultations, not taken into account in this study, could have an influence on the overall experience, level of satisfaction and willingness of future use of TM consultation. All these limitations justify a future study, at a different period (post-COVID-19 pandemic) with a more representative sample, using a face-to-face data collection approach. Triangulating information from the perspectives of service providers and users, using mixed methods studies, would provide a comprehensive identification of the gaps to be addressed for the efficient and sustainable integration of this emerging mode of health care delivery.

## Conclusion

TM consultations are an emerging pertinent complement to support the conventional consultation formats in the future. To ensure its sustainability technology must be augmented to provide a greater level of security that guarantees privacy for users and offer an experience comparable to face-face consultations.

## Data availability statement

The raw data supporting the conclusions of this article will be made available by the authors, without undue reservation.

## Ethics statement

The studies involving humans were approved by the Research and Ethics Committee of the College of Medicine and Medical Sciences at Arabian Gulf University (approval number: E17-PI-11–21) and the primary healthcare research and ethics committee. The studies were conducted in accordance with the local legislation and institutional requirements. Written informed consent for participation was not required from the participants or the participants' legal guardians/next of kin because data was collected through phone interviews and participants were provided information about the study and informed that participation was entirely voluntary and that refusing to participate would not affect future services. Prior to enrolling, all respondents provided informed consent by phone, which was documented by the interviewer in the consent form.

## Author contributions

FHab: Conceptualization, Data curation, Formal analysis, Investigation, Methodology, Software, Supervision, Validation, Visualization, Writing—original draft, Writing—review and editing. AR: Investigation, Writing—original draft. ZH: Investigation, Writing—original draft. HAl: Investigation, Writing—original draft. JA: Investigation, Writing—original draft. FHay: Investigation, Writing—original draft. AA: Data curation, Formal analysis, Writing—original draft. HAb: Investigation, Writing—original draft. AB: Conceptualization, Investigation, Methodology, Project administration, Supervision, Validation, Writing—review and editing, Writing—original draft.
